# ^68^Ga-PSMA-PET/CT for the evaluation of liver metastases in patients with prostate cancer

**DOI:** 10.1186/s40644-019-0220-x

**Published:** 2019-06-11

**Authors:** Jonathan Damjanovic, Jan-Carlo Janssen, Vikas Prasad, Gerd Diederichs, Thula Walter, Winfried Brenner, Marcus R. Makowski

**Affiliations:** 1 Department of Radiology, Charité, Charitéplatz 1, 10117 Berlin, Germany; 2 Department of Nuclear Medicine, University of Ulm, Albert-Einstein-Allee 23, 89081 Ulm, Germany; 3 Department of Nuclear Medicine, Charité, Augustenburger Platz 1, 13353 Berlin, Germany; 4 Division of Imaging Sciences, King’s College London, London, England

**Keywords:** Liver metastasis, PSMA, PET/CT, Prostate cancer

## Abstract

**Background:**

The purpose of this study was to evaluate the imaging properties of hepatic metastases in ^68^Ga-PSMA positron emission tomography (PET) in patients with prostate cancer (PC).

**Methods:**

^68^Ga-PSMA-PET/CT scans of PC patients available in our database were evaluated retrospectively for liver metastases. Metastases were identified using ^68^Ga-PSMA-PET, CT, MRI and follow-up scans. Different parameters including, maximum standardized uptake values (SUV_max_) of the healthy liver and liver metastases were assessed by two- and three-dimensional regions of interest (2D/3D ROI).

**Results:**

One hundred three liver metastases in 18 of 739 PC patients were identified. In total, 80 PSMA-positive (77.7%) and 23 PSMA-negative (22.3%) metastases were identified. The mean SUV_max_ of PSMA-positive liver metastases was significantly higher than that of the normal liver tissue in both 2D and 3D ROI (*p* ≤ 0.05). The mean SUV_max_ of PSMA-positive metastases was 9.84 ± 4.94 in 2D ROI and 10.27 ± 5.28 in 3D ROI; the mean SUV_max_ of PSMA-negative metastases was 3.25 ± 1.81 in 2D ROI and 3.40 ± 1.78 in 3D ROI, and significantly lower than that of the normal liver tissue (*p* ≤ 0.05). A significant (p ≤ 0.05) correlation between SUV_max_ in PSMA-positive liver metastases and both size (ρ_Spearman_ = 0.57) of metastases and PSA serum level (ρ_Spearman_ = 0.60) was found.

**Conclusions:**

In ^68^Ga-PSMA-PET, the majority of liver metastases highly overexpress PSMA and is therefore directly detectable. For the analysis of PET images, it has to be taken into account that also a significant portion of metastases can only be detected indirectly, as these metastases are PSMA-negative.

## Background

Worldwide, prostate cancer (PC) is considered the second most frequently diagnosed cancer in men and the fifth leading cause of cancer death [1]. Recently, radiolabeled prostate-specific membrane antigen (PSMA) ligands such as ^68^Ga-PSMA-HBED-CC have been introduced as a promising radiotracer for the PET imaging of PC [2]. PSMA is a transmembrane protein that is significantly overexpressed in most prostate cancer cells [3]. Different studies demonstrated that ^68^Ga-PSMA–PET enables imaging with a higher specificity and sensitivity regarding the detection of metastases, compared to current standard imaging (CT, MRI and bone scintigraphy) and other PET tracers such as ^18^F-Choline [4–7]. It also improves detection of metastatic lesions at low serum PSA levels in biochemically recurrent prostate cancer [8].

The liver is considered to be the third most common site for systemic metastases in PC (25%), after bone (90%) and lung (46%), according to autopsy studies [9]. The prevalence of clinical liver metastases in retrospective studies was 4.3 and 8.0% [10, 11]. Liver metastases typically occur in systemic, late stage, hormone refractory disease [10]. However, there are reports of patients with liver metastases as the first site of metastatic disease and the liver representing the only metastatic site [10, 12, 13]. Especially in this patient collective, early and reliable detection of liver metastases is of high clinical importance for accurate staging and therapy planning.

There is evidence that in PC, liver metastases are frequently associated with neuroendocrine characteristics; in a prospective study of 28 patients with liver metastases, Pouessel et al. measured increased levels of the neuroendocrine serum markers chromogranin A and neurone-specific enolase in 84 and 44% of the patients, and out of six patients with a pathological analysis, two had neuroendocrine metastases [10]. Neuroendocrine transdifferentiation might lead to loss of PSMA-expression and therefore impede the visualization of liver metastases in ^68^Ga-PSMA-PET [14]. Furthermore, the relatively high background activity of the liver might also affect the visibility of liver metastases in ^68^Ga-PSMA-PET [14]. Imaging of hepatic PC metastases in ^68^Ga-PSMA-PET has been reported in case reports, but not been systematically researched in a larger cohort of patients [12, 15–18].

Therefore, the aim of this study was to investigate the ^68^Ga-PSMA-PET imaging properties of liver metastases in PC patients.

## Methods

### Study population

For this retrospective study, we obtained approval from our institutional ethics review board. We extracted 739 consecutive patients with confirmed prostate cancer from our local database who underwent at least one ^68^Ga-PSMA-PET/CT between September 2013 and April 2017. Out of these, we identified eighteen patients with liver metastases, according to the criteria described below. Prostate cancer was histologically proven in all patients. Only patients with no other known type of cancer but PC were included. All available additional information from clinical records were obtained. Patients’ characteristics are summarized in Table 1. Gleason score (GS) was available in eleven, therapy information only in thirteen and PSA level only in twelve patients.

**Table 1:** Characteristics of the study collective of PC patients with liver metastases

Characteristic	Mean ± SD	Median (Range)	*n* (%)
Age (years)	70.1 ± 8.5	71.0 (54.5–81.4)	
PSA (ng/ml)	556.3 ± 1398.4	124.6 (0.01–4962.0)	
Gleason score		9 (6–10)	
Therapy			13
RP			7 (53.8)
RT			6 (46.2)
ADT			11 (84.6)
CTX			7 (53.8)
^177^Lu-PSMA			4 (30.8)

*RP* Radical prostatectomy, *RT* Radiotherapy, *ADT* Androgen deprivation therapy, *CTX* Chemotherapy

Summary of the patients’ characteristics, including age, PSA, GS, indication for imaging and previous therapy, at the time imaging was performed. *GS* Gleason score, *PSA* prostate-specific antigen

### Positron emission tomography tracer

^68^Ga was eluted from a conventional ^68^Ge/^68^Ga radionuclide generator (Eckert & Ziegler Radiopharma GmbH, Berlin, Germany) and compounded with PSMA-HBED-CC (ABX GmbH, Radeberg, Germany) according to the method described previously [19, 20].

### Imaging protocol

PET/CT imaging was performed 75.8 ± 18.2 min after intravenous injection of 120.5 ± 25.7 MBq of ^68^Ga-PSMA. PET scans were acquired using a Gemini Astonish TF 16 PET/CT scanner (Phillips Medical Systems) in 3D acquisition mode [21]. Axial, sagittal and coronal slices were reconstructed (144 voxels with 4mm^3^, isotropic). Before PET scan, a low-dose CT was performed for anatomical mapping and attenuation correction (30 mAs, 120 kVp). Each bed position was acquired for 1.5 min with a 50% overlap. In case contrast-enhanced CT (CE-CT) was performed, 80–120 ml of contrast agent (Ultravist® 370, Bayer Schering Pharma, Berlin, Germany) was injected intravenously with a delay of 70 s for the venous phase.

### Imaging analysis

Two experienced observers analyzed the PET/CT scans using Visage 7.1 (Visage Imaging GmbH, Berlin, Germany). For the diagnosis of metastases, all available imaging studies including all imaging modalities (CT, MRI, ^68^Ga-PET) of the patients were taken into consideration. At least two of the following four criteria had to be fulfilled for the diagnosis of liver metastasis: (I) CT imaging with low-to-isoattenuating masses [22]; (II) MRI with typical presentation of liver metastases according to guidelines [23]; (III) high focal uptake of ^68^Ga-PSMA in PET distinctively above normal heterogeneity; (IV) new appearance or significant change of size of lesions according to the RECIST 1.1 criteria compared to previous studies within the same modality with a minimum follow-up interval of six months [24]. Patients with signs of a malignancy other than PC were excluded. Out of 23 patients with suspected liver metastases, five patients dropped out because they did not fulfill these criteria. Overall 18 patients with hepatic metastases were identified out of 739 patients. Among these, criteria I was fulfilled by all patients, criteria II by four patients, criteria III by 16 patients and criteria IV by 12 patients. Maximum ten metastases per patient were analyzed. In case a patient was imaged more than once, only the most recent ^68^Ga-PSMA-PET scan was included in this study. As a result, 103 liver metastases were analyzed as part of this study. The sizes of metastases were measured based on the CT scan. Regarding the evaluation of the radiodensity, two groups were formed. One group in which only unenhanced CTs were available (five patients) and another group in which contrast-enhanced CTs were available (13 patients).

To normalize standardized uptake values (SUV) for body weight, they were calculated by the software using with the equation *SUV* = *C*_*tis*_/*Q*_*inj*_/*BW*, where *C*_*tis*_ is the lesion activity concentration in MBq per milliliter, *Q*_*inj*_ is the activity injected in MBq, and *BW* is the bodyweight in kilograms. For PET data quantification, a two-dimensional region of interest (2D ROI), as well as a three-dimensional region of interest (3D ROI), were defined. ^68^Ga-PSMA-HBED-CC uptake was quantified using maximum standardized uptake values (SUV_max_). All values were recorded in the transaxial, attenuation-corrected PET-slice representing the greatest extent of the respective lesion. Regions of interest were defined manually in freehand mode avoiding the periphery of lesions to minimize partial volume effects. SUV_max_ of the healthy liver was measured in a region with minimal irregularities. An SUV_max_-lesion-to-background ratio (LBR) was calculated for all metastases in 3D ROI, using the formula $LBR=\frac{SUV_{max}\  of\ metastasis}{SUV_{max}\  of\ liver}$. Any tracer uptake 20% or more above liver uptake was considered PSMA-positive, any tracer uptake below that was considered PSMA-negative. The readers were blinded to the results of other diagnostic procedures and the clinical history of the patients.

### Statistical analysis

The descriptive statistics are reported as mean, median and/or range when applicable. Nonparametric statistical tests were used as the data contained several outliers. The Mann-Whitney *U* test was used for the comparison of SUV_max_ values and mean radiodensity values (HU_mean_) between the healthy liver and liver metastases. SUV_max_ values in 2D and 3D ROI were compared using the Wilcoxon signed-rank test. To determine the relationship between SUV_max_ and size of lesions, patients’ age and PSA serum level, a Spearman’s rank correlation was used. A binomial test was run to evaluate the distribution of liver metastases among the hepatic lobes. The significance level was set to α < 0.05. Statistical analyses were conducted with SPSS 23 for Mac (IBM Corp, Armonk, NY).

## Results

### Characteristics of the study patients

In total, 103 liver metastases were detected in 18 of 739 (2.44%) patients. Patients’ characteristics are summarized in Table 1. Mean patients’ age was 70.1 ± 8.5 years. Median GS was 9 (range 6–10). Mean PSA level was 556.3 ± 1398.4 ng/ml.

### Lesion-based analysis of liver metastases

**Fig. 1:**
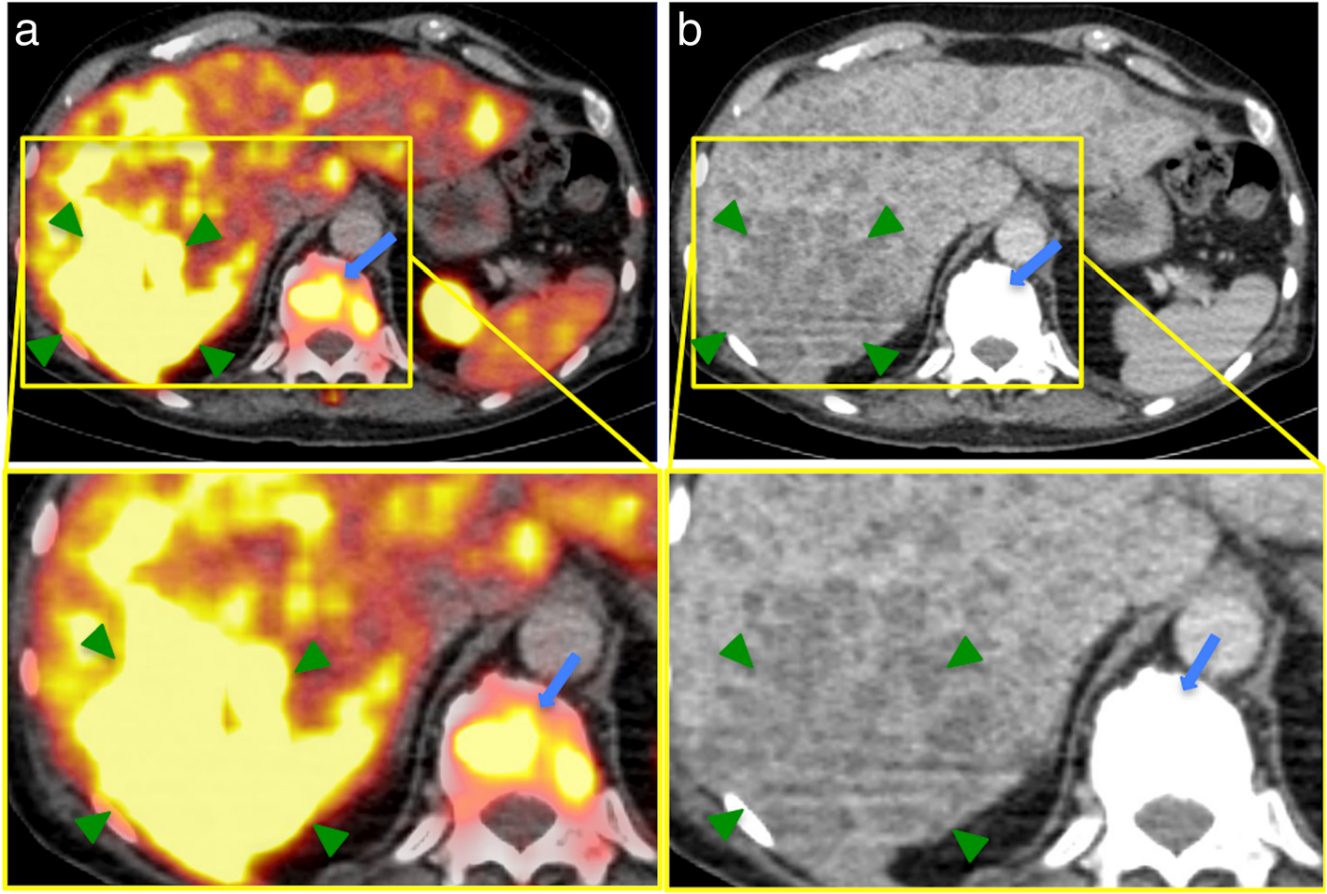
Example of ^68^Ga-PSMA-positive liver metastases in a PC patient with a recurrent acinar adenocarcinoma. **a**, **b**: ^68^Ga-PSMA-PET/CT of a 68-year-old patient with a recurrent acinar adenocarcinoma of the prostate. At initial diagnosis in 2007, the GS was 5 + 5. The patient received primary radiotherapy and undergone chemotherapy as well as androgen-deprivation therapy. The serum PSA was 606 ng/ml at the time of examination. Besides disseminated osseous metastases (such as in a vertebral body, blue arrows) and a singular lymph node metastasis in the axilla, the PET/CT (**a)** revealed small-nodular, PSMA-positive liver metastases in all segments, with SUV_max_-values up to 26.3 (exemplary in segments VII/VIII, green arrows). In contrast-enhanced CT (**b**), liver metastases appear ill-defined and hypodense compared to the liver, typical for hypovascular metastases. *GS* Gleason score, *PSA* prostate-specific antigen, *SUV*_*max*_ Maximum standardized uptake value

**Fig. 2:**
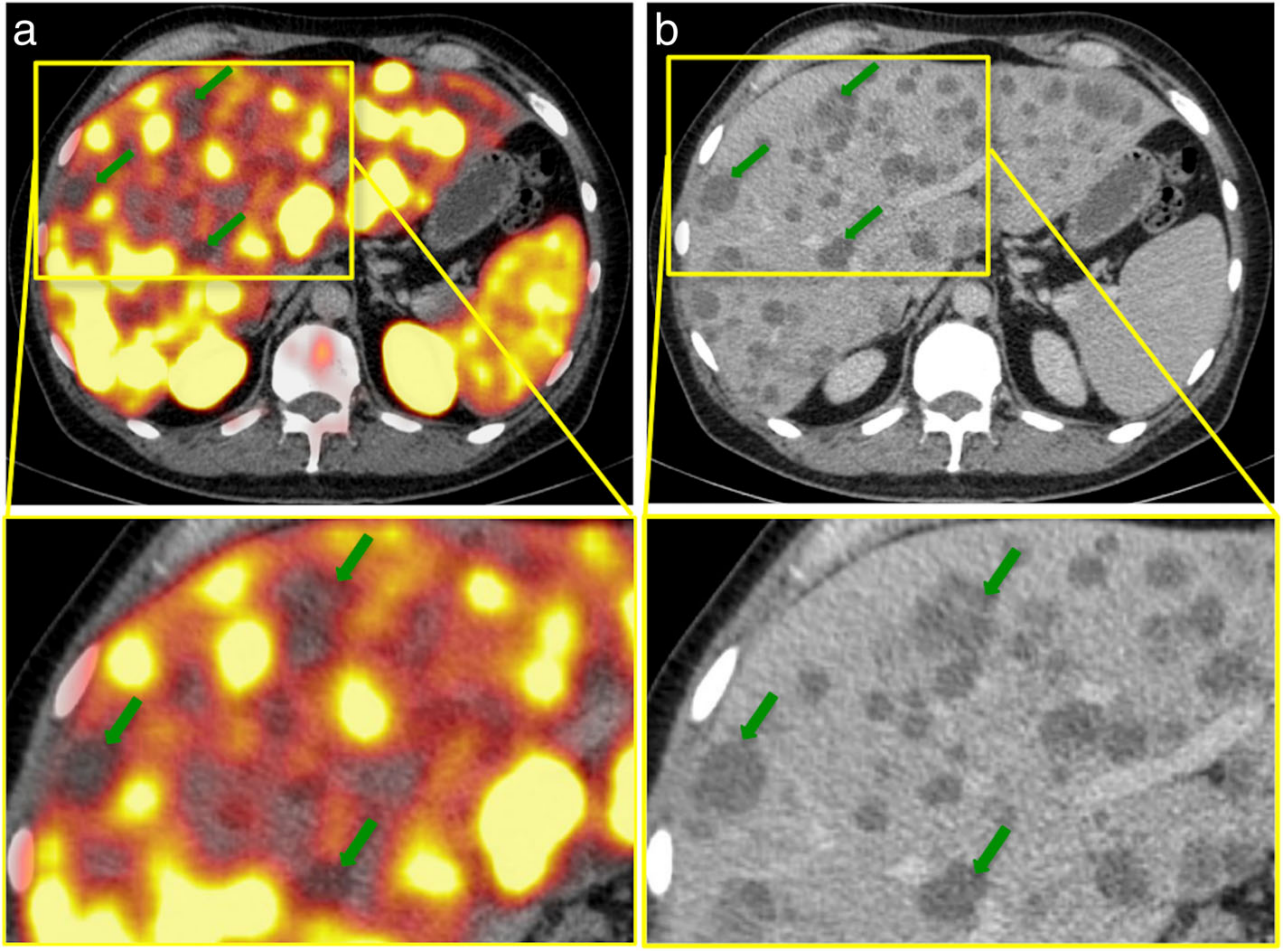
Example of ^68^Ga-PSMA-negative liver metastases in a PC patient with a recurrent adenocarcinoma**. a**, **b**: ^68^Ga-PSMA-PET/CT of a 54-year-old patient with a recurrent adenocarcinoma of the prostate and disseminated lymph node, bone, and hepatic metastases. After the initial diagnosis in 2011, the patient had received a radical prostatectomy and undergone chemotherapy as well as androgen-deprivation therapy. The serum PSA was 4962.0 ng/ml at the time of examination; the initial GS was 4 + 5. The PET/CT (**a**) illustrates disseminated, PSMA-negative liver metastases, with SUV_max_-values up to 4.2 (liver background 9.5). Green arrows point to examples of liver metastases in segments IVa and V. In CE-CT (**b**), liver metastases appear hypodense compared to the liver. *GS* Gleason score, *PSA* prostate-specific antigen, *SUV*_*max*_ Maximum standardized uptake value

**Fig. 3:**
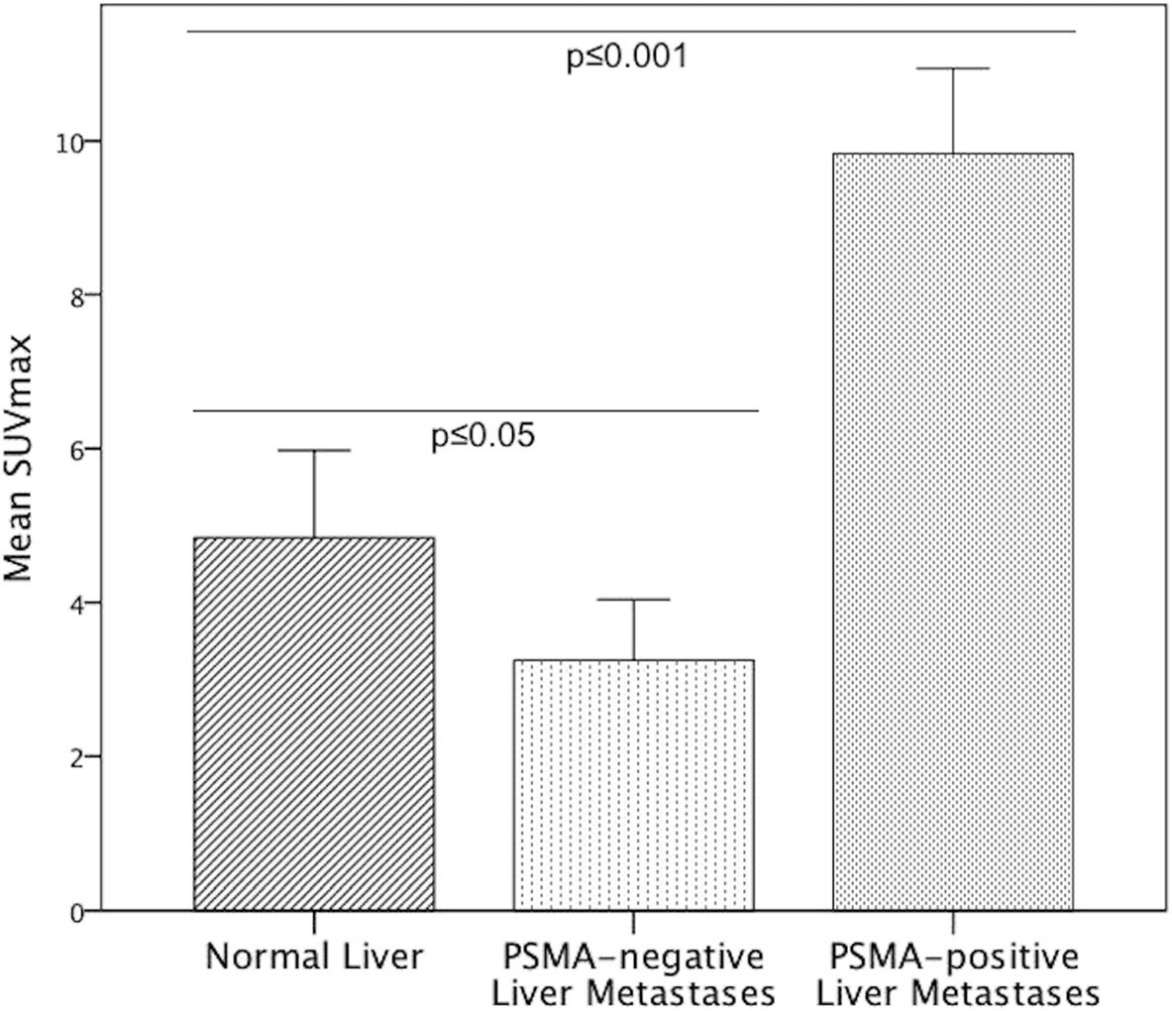
SUV_max_ of the normal liver, PSMA-positive and PSMA-negative liver metastases in 2D ROI. The mean SUV_max_ of PSMA-positive liver metastases was 9.8 ± 4.9 and significantly higher than the mean SUV_max_ of the normal liver (4.8 ± 2.3, *p* ≤ 0.001). In contrast, the mean SUV_max_ of PSMA-negative liver metastases was 3.3 ± 1.8 and significantly lower than that of the normal liver (*p* ≤ 0.05). *SUV*_*max*_ Maximum standardized uptake value

**Fig. 4:**
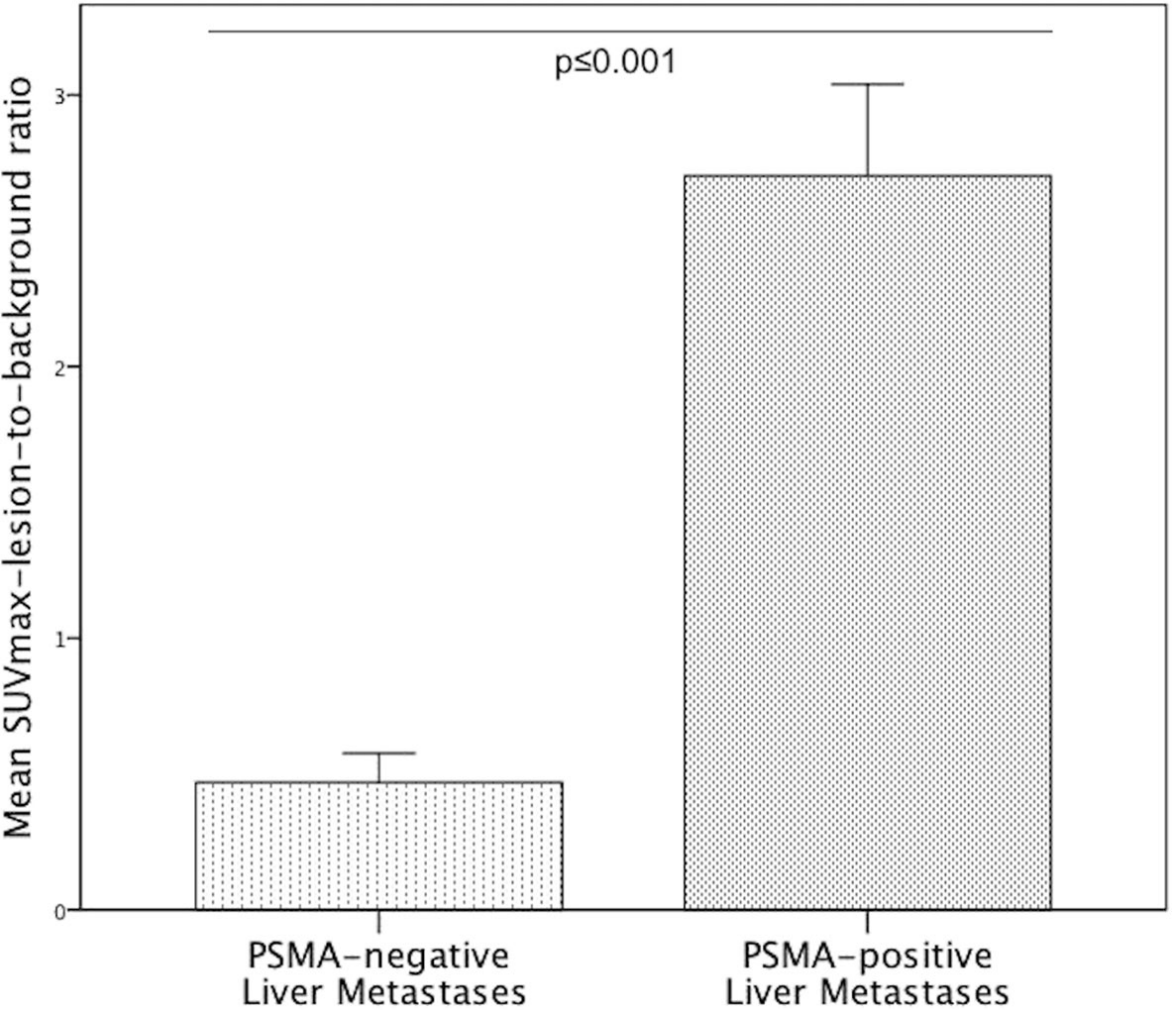
Mean SUV_max_-lesion-to-background ratio of PSMA-positive and PSMA-negative liver metastases. The mean SUV_max_-lesion-to-background ratio in PSMA-positive liver metastases was 2.7 ± 1.5 and significantly higher than that of PSMA-negative metastases (0.5 ± 0.3, p ≤ 0.001). *SUV*_*max*_ Maximum standardized uptake value

All detailed results are depicted in Table 2. The mean size of metastases was 3.3 ± 4.7 cm^2^ (range 0.2–29.5cm^2^). The mean SUV_max_ of all liver metastases was 8.4 ± 5.2 in 2D and 8.7 ± 5.5 in 3D ROI, compared to a mean SUV_max_ of the normal liver of 4.8 ± 2.3 in 2D and 5.3 ± 2.3 in 3D ROI. The mean SUV_max_ of all liver metastases was significantly higher than the SUV_max_ of normal liver in both 2D (*p* ≤ 0.05) and 3D ROI (p ≤ 0.05). In total, 80 PSMA positive (77.7%) and 23 PSMA negative (22.3%) metastases were identified. Examples of PSMA-positive and PSMA-negative metastases are illustrated in Figs. 1 and 2. The mean SUV_max_ of PSMA-positive metastases was 9.8 ± 4.9 in 2D (see Fig. 3) and 10.3 ± 5.3 in 3D ROI. The mean SUV_max_ of PSMA-negative metastases was 3.3 ± 1.8 in 2D and 3.4 ± 1.8 in 3D ROI. This was significantly lower than the mean SUV_max_ of the normal liver, in both 2D (*p* ≤ 0.05) and 3D ROI (*p* ≤ 0.001). The mean SUV_max_ obtained by 3D ROI was significantly higher than that obtained by 2D ROI in normal liver (p ≤ 0.05) as well as in PSMA-positive liver metastases (*p* ≤ 0.001). There was no difference in SUV_max_ of PSMA-negative metastases between 2D and 3D ROI (*p* > 0.05). The mean SUV_max_-lesion-to-background ratio in PSMA-positive liver metastases was 2.7 ± 1.5, which was significantly higher than that of PSMA-negative metastases (0.5 ± 0.3, *p* ≤ 0.001, see Fig. 4).

**Table 2:** Comparison of size, ^68^Ga-PSMA-HBED-CC uptake (SUV_max_) and radiodensity (HU_mean_) between normal liver and liver metastases (all, PSMA-positive and PSMA-negative)

	Normal liver	All liver metastases	PSMA positive metastases	PSMA negative metastases	*p*-value
Number	18	103	80	23	
Size in cm^2^		3.27 ± 4.73 (0.2–29.5)	2.69 ± 4.95 (0.2–29.5)	5.29 ± 3.23 (2.1–14.3)	
SUV_max_ 2D ROI	4.84 ± 2.29 (2.9–10.7)	8.37 ± 5.22 (1.0–26.3)			≤0.05
			9.84 ± 4.94 (3.6–26.3)		≤0.001
				3.25 ± 1.81 (1.0–7.5)	≤0.05
SUV_max_ 3D ROI	5.32 ± 2.28 (3.0–11.9)	8.73 ± 5.53 (1.4–26.3)			≤0.05
			10.27 ± 5.28 (3.6–26.3)		≤0.001
				3.40 ± 1.78 (1.4–7.8)	≤0.001
HU_mean_, CE-CT	102.18 ± 17.09 (56.5–124.0)	61.04 ± 25.10 (16.4–124.2)			≤0.001
			67.0 ± 21.49 (16.5–124.2)		≤0.001
				30.35 ± 19.71 (16.4–65.0)	≤0.001
HU_mean_, unenhanced CT	53.76 ± 8.89 (38.2–60.6)	31.10 ± 13.94 (8.5–50.7)			≤0.05
			40.36 ± 11.05 (8.5–50.7)		≤0.05
				19.07 ± 5.27 (13.1–26.5)	≤0.05

All data are given as mean ± standard deviation and range in parentheses. *SUV*_*max*_ Maximum standardized uptake value, *ROI* Region of interest, *HU*_*mean*_ Mean Hounsfield units, *CE-CT* Contrast-enhanced CT

The mean SUV_max_ of all liver metastases was significantly higher than the SUV_max_ of the normal liver, both in 2D (*p* ≤ 0.05) and 3D ROI (*p* ≤ 0.05). The mean SUV_max_ of PSMA-negative liver metastases was significantly lower than the SUV_max_ of the normal liver, in 2D (*p* ≤ 0.05) and 3D ROI (*p* ≤ 0.001). The mean CT attenuation value HU_mean_ of PSMA-positive metastases was significantly lower than that of normal liver, in contrast-enhanced (*p* ≤ 0.001) as well as in unenhanced CT (*p* ≤ 0.05). *SUV*_*max*_ Maximum standardized uptake value, *ROI* Region of interest, *HU*_*mean*_ Mean Hounsfield units

### HU_mean_ of liver metastases compared to the normal liver

The mean CT attenuation value of liver metastases was significantly lower than that of the normal liver, in CE-CT (p ≤ 0.001) and unenhanced CT (*p* ≤ 0.05). In liver metastases, HU_mean_ was 61.0 ± 25.1 in CE-CT and 31.1 ± 13.9 in unenhanced CT, whereas the HU_mean_ of the normal liver was 102.2 ± 17.1 in CE-CT and 53.8 ± 8.9 in unenhanced CT. In PSMA-negative metastases, HU_mean_ was 30.4 ± 19.7 in CE-CT and 19.1 ± 5.3 in unenhanced CT. In PSMA-positive metastases, HU_mean_ was 67.0 ± 21.5 in CE-CT and 40.4 ± 11.1 in unenhanced CT. HU_mean_ of PSMA-negative metastases was found to be significantly lower than that of PSMA-positive metastases, in both contrast-enhanced and unenhanced CT (both *p* ≤ 0.001).

### Correlation between size and SUV_max_ of liver metastases

**Fig. 5:**
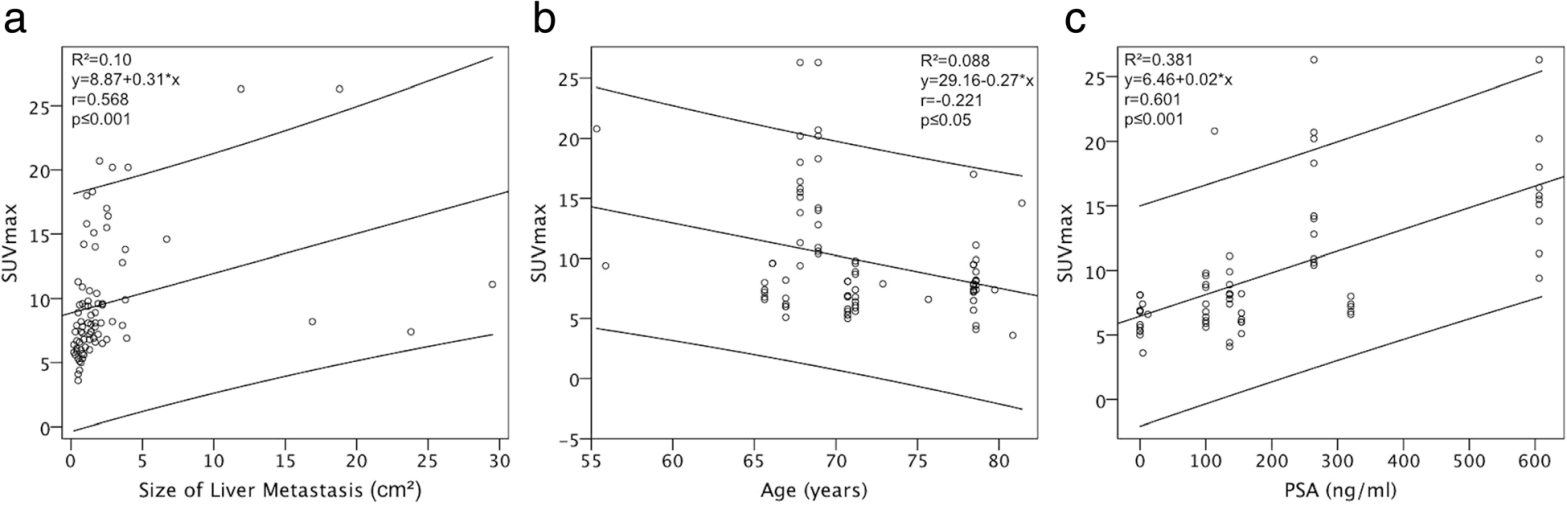
Correlations between metastases’ size, patients’ age, serum PSA and SUV_max_ of metastases. **a-c**: Correlations according to a Spearman’s correlation, including 95% confidence intervals. A moderate significant association between the SUV_max_ in liver metastasis and its size (**a**) was calculated (p ≤ 0.001, ρ_Spearman_ = 0.568, 95% CI [0.397, 0.701]). Patient’s age and SUV_max_ of liver metastases (**b**) weakly correlate (p ≤ 0.05, ρ_Spearman_ = − 0.221, 95% CI [− 0.420; − 0.002]). Serum PSA and SUV_max_ of liver metastases (**c**) moderately correlate (p ≤ 0.001, ρ_Spearman_ = 0.601, 95% CI [0.419; 0.736]). *R*^*2*^ Coefficient of determination, *r* Spearman’s rho, *SUV*_*max*_ Maximum standardized uptake value, *PSA* Prostate-specific antigen

We calculated a moderate significant positive relationship between size and SUV_max_ of PSMA-positive metastases (Fig. 5a, ρ_Spearman_ = 0.568, 95% CI [0.397; 0.701], *p* ≤ 0.001).

### Patient-based analysis and correlation between PSA, patients’ age, and SUV_max_

**Fig. 6:**
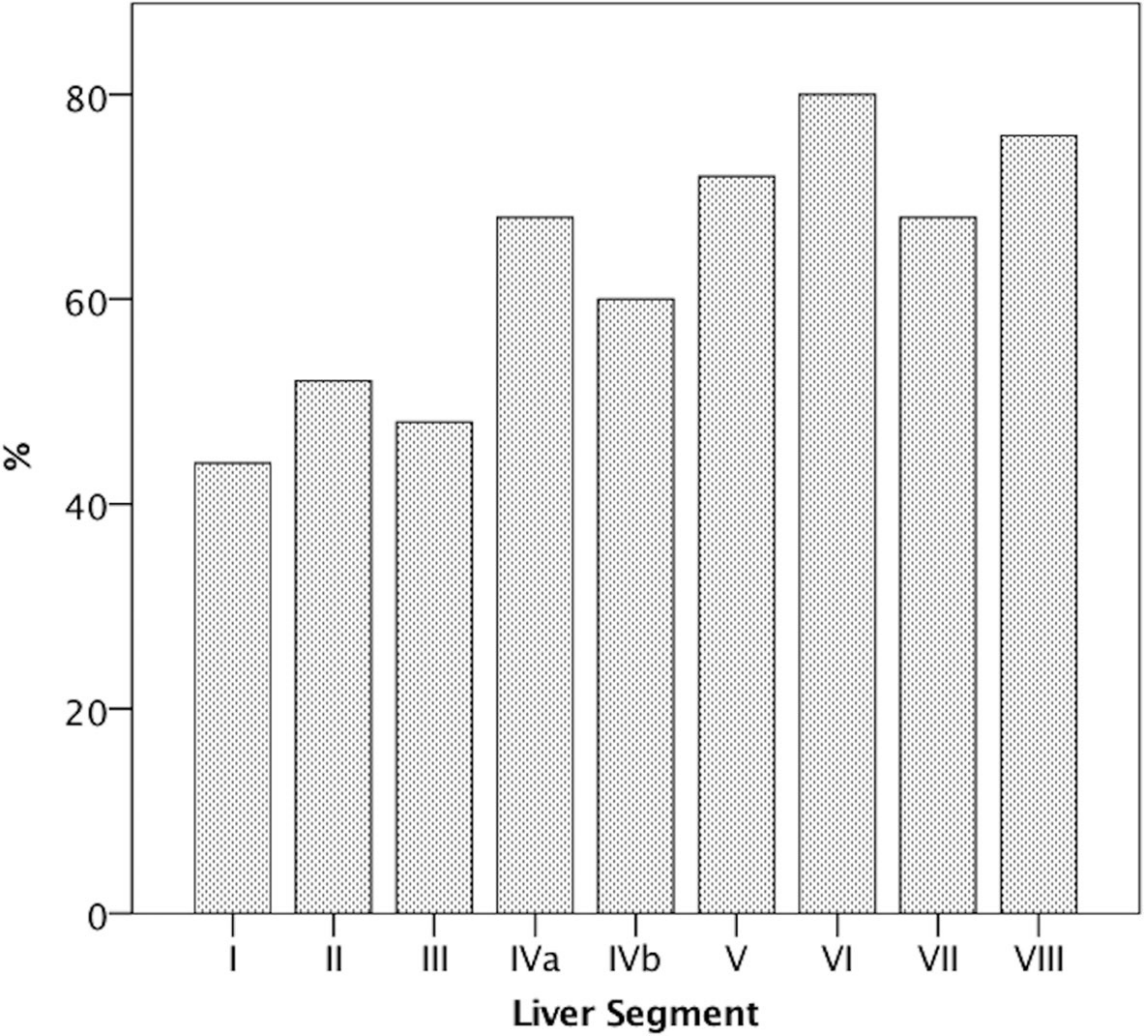
Patient-based analysis of the localization of liver metastases, according to liver segments. Percentages indicate the proportion of study patients in whom liver metastases were localized within the respective liver segment. Liver segment VI was the most common localization for liver metastases (80%), whereas liver segment I was the least common site (44%)

Of 18 patients with liver metastases, eight patients (44,4%) had ten or more metastases, three patients (16.7%) had two to ten metastases, and seven patients (38.9%) had a single metastasis. Regarding the tracer uptake, 15 patients (83.3%) had PSMA-positive hepatic metastases only, two patients (11.1%) had PSMA-negative metastases only, and one patient (5.6%) had mixed metastases. The distribution of liver metastases by liver segments is illustrated in Fig. 6. A higher number of patients had liver metastases in the right (100%) than in the in the left hepatic lobe (61.1%, *p* > 0.05). A weak, significant negative relationship between patients’ age and SUV_max_ of PSMA-positive metastases was calculated (Fig. 5b, ρ_Spearman_ = − 0.221, 95% CI [− 0.420; − 0.002], *p* ≤ 0.05). Also, there was a moderate, significant positive correlation between the PSA serum level at the time of examination and SUV_max_ of PSMA-positive metastases (Fig. 5c, ρ_Spearman_ = 0.601, 95% CI [0.419; 0.736], *p* ≤ 0.001).

## Discussion

This study evaluated the imaging characteristics of liver metastases in ^68^Ga-PSMA-PET. It was demonstrated that the majority of liver metastases highly overexpress PSMA and is therefore directly detectable by ^68^Ga-PSMA-PET. For the analysis of PET images, it has to be taken into account that also a significant portion of metastases can only be detected indirectly, as these metastases are PSMA-negative.

^68^Ga-PSMA-PET/CT has demonstrated potential to improve the initial staging, lymph node staging, and detection of recurrence of PC, even at low PSA levels. Several studies have indicated that ^68^Ga-PSMA-PET is more accurate compared to other tracers as such as ^18^F-choline [25]. So far, the imaging properties of liver metastases in ^68^Ga-PSMA-PET have not been systematically researched.

In our cohort, liver metastases were present in 2.4% of patients who underwent ^68^Ga-PSMA-PET. This was lower compared to the prevalence reported by other studies, likely as a result of the different study designs and the limited sensitivity of PET for the detection of small (< 1 cm) metastases [10, 11]. In our study population, the majority of patients demonstrated PSMA-positive hepatic metastases, while only a small number of patients demonstrated PSMA-negative or mixed metastases. An explanation for the difference of ^68^Ga-PSMA-HBED-CC uptake in liver metastases could be the diversity of phenotypes in metastases, predominantly the neuroendocrine trans-differentiation. In PC, liver metastases are frequently associated with neuroendocrine characteristics as well as with advanced state in systemic disease [10]. It is thought that the degree of neuroendocrine trans-differentiation increases with disease progression and in response to ADT [26]. A pronounced elevation of neuroendocrine serum markers such as neuron-specific enolase and chromogranin A has been demonstrated in patients with long duration of ADT [27]. Autopsy studies have confirmed the phenotypic heterogeneity of end-stage metastatic prostate cancer [28, 29]. A large part of neuroendocrine prostate cancer cells does not express generic PC biomarkers including P501S, PSMA, and PSA [30]. This is consistent with the histopathologic finding in one of our study patients with PSMA-negative liver metastases, in whom liver and prostate biopsy were performed. Histopathology of the metastasis revealed an infiltration of the liver with neuroendocrine carcinoma cells, which were positive for the neuroendocrine biomarker CD56, but negative for PSA, PSMA and androgen receptor. In the same patient, histopathology of the prostate tissue exposed an acinar adenocarcinoma with 5% of the cells presenting neuroendocrine markers, which can be interpreted as a partial trans-differentiation. The findings of this study are also consistent with a case report by Usmani et al. of a PC patient with an unsuspicious ^68^Ga-PSMA-PET, whereas a ^68^Ga-DOTANOC-PET performed ten days later revealed multiple somatostatin-avid hepatic and lymph node metastases, and lymph node cytology confirmed neuroendocrine differentiation [31]. Overall, neuroendocrine trans-differentiation could explain the loss of PSMA-expression of liver metastases in progressive disease. Vice versa, the detection of PSMA-underexpression in liver metastases could represent trans-differentiation; clinicians need to be familiar with this concept as it may result in treatment adaptation.

Interestingly, the radiodensity of PSMA-negative liver metastases was significantly lower compared to the PSMA-positive metastases, in both unenhanced and contrast-enhanced CTs. This finding could further support the differentiation of liver metastases in PC but needs to be verified in a larger cohort.

Additionally, a significant positive correlation between the serum PSA level at the time of examination and SUV_max_ of PSMA-positive liver metastases was observed. This could be explained by the fact that both parameters tend to increase within the progression of the disease. The finding is consistent with the studies of Koerber et al. and Sachkepides et al., who reported that patients with higher PSA values demonstrated a significant higher tracer uptake in intraprostatic tumor lesions on PSMA-PET/CTs [32, 33]. Between the size and SUV_max_ of PSMA-positive liver metastases, a weak but significant association was found. This might be the result of a proliferative advantage of highly PSMA-expressing cells, as it has been demonstrated in-vitro [34]. We further observed a weak but significant, negative association between age and SUV_max_ of PSMA-positive liver metastases. A hypothesis explaining this finding could be that patients who develop liver metastases at a younger age have a more aggressive subtype of PC with higher PSMA-expression. This, however, needs to be investigated in a larger cohort.

A limitation of this retrospective study is that diagnoses of liver metastases were not confirmed histopathologically since no biopsies of most of the metastases were performed. A possible limitation to the lesion-based analysis regarding the calculation of mean SUV_max_ values could be due to an overestimation of the patients subgroup with multiple metastases compared to the subgroup with few metastases.

## Conclusions

The majority of liver metastases highly overexpress PSMA in ^68^Ga-PSMA-PET and is therefore directly detectable. For the analysis of PET images, it has to be taken into account that also a significant portion of metastases can only be detected indirectly, as these metastases are PSMA-negative. Future studies are warranted to test these findings in a larger collective of patients and to correlate changes on histopathology with the PSMA expression.

## Data Availability

The datasets used and/or analyzed during the current study are available from the corresponding author on reasonable request.

## Abbreviations

ADT: Androgen deprivation therapy
CE-CT: Contrast-enhanced CT
CTX: Chemotherapy
Ga: Gallium
GS: Gleason score
HU: Hounsfield unit
PC: Prostate cancer
PET/CT: Positron emission tomography / computed tomography
PSA: Prostate-specific antigen
PSMA: Prostate specific membrane antigen
ROI: Region of interest
RP: Radical prostatectomy
RT: Radiotherapy
SUV: Standardized uptake value
